# Interaction rates, vital rates, background fitness and replicator dynamics: how to embed evolutionary game structure into realistic population dynamics

**DOI:** 10.1007/s12064-017-0257-y

**Published:** 2017-11-20

**Authors:** K. Argasinski, M. Broom

**Affiliations:** 1 Institute of Mathematics of Polish Academy of Sciences, ul. Śniadeckich 8, 00-956 Warszawa 10, Poland; 2 Department of Mathematics, City, University of London, Northampton Square, London, EC1V 0HB UK

**Keywords:** Replicator dynamics, Evolutionary game, Density dependence, Interaction rate, Eco evolutionary feedback, Background fitness, 91A22, 92D15, 37C10

## Abstract

In this paper we are concerned with how aggregated outcomes of individual behaviours, during interactions with other individuals (games) or with environmental factors, determine the vital rates constituting the growth rate of the population. This approach needs additional elements, namely the rates of event occurrence (interaction rates). Interaction rates describe the distribution of the interaction events in time, which seriously affects the population dynamics, as is shown in this paper. This leads to the model of a population of individuals playing different games, where focal game affected by the considered trait can be extracted from the general model, and the impact on the dynamics of other events (which is not neutral) can be described by an average background fertility and mortality. This leads to a distinction between two types of background fitness, strategically neutral elements of the focal games (correlated with the focal game events) and the aggregated outcomes of other interactions (independent of the focal game). The new approach is useful for clarification of the biological meaning of concepts such as weak selection. Results are illustrated by a Hawk–Dove example.

## Introduction

The cornerstone of building scientific theories is the proper choice of underlying terminology describing the objects and processes of interest; the mathematical structures used in the formalization of the theory can influence the underlying language. A good example is the impact of game theory on evolutionary theory, which has meant that strategic reasoning is common in works related to evolution, even if they are not supported by mathematical notions (Dawkins 1976; Williams 1996). However, the basic evolutionary game theoretic framework is described by abstract mathematical terms whose relations with observable biological processes is often unclear. The most influential concept, which is foundational for game theoretic methods in biology, is the game as a metaphor for the individual interactions. In this paper we will investigate how this aspect should be expressed in the context of the ecological population dynamics.

The modern approaches to evolutionary game modelling can essentially be divided into two classes. The first contains static models (see e.g. Maynard Smith 1982; Broom and Rychtar 2013), based on potentially complicated payoff functions describing some abstract parameter called “fitness”, while the second contains dynamic models based on replicator dynamics and simplified (mostly matrix) payoff functions (Maynard Smith 1982; Cressman et al. 1986; Hofbauer and Sigmund 1988, 1998). The first type is focused on the details of the interaction, while population dynamics aspects are lacking. There are no evolutionary processes in time, only causal outcomes of the particular interaction. Thus, while payoffs quantified by obtained resources or energetic gain have a clear biological interpretation, the impact of the game outcomes on the population state is not fully explained. In the second case, the situation is the opposite: the interactions are not explicitly depicted in the model but their outcomes are phenomenologically described by the excess from the average growth rate, and the dynamics of the selection process is explicitly analysed. In addition, from game theoretic methods have grown the field of adaptive dynamics (Dieckmann and Law 1996; Metz et al. 1996; Geritz et al. 1998; Dercole and Rinaldi 2008), focused on the long-term evolution of continuous traits, driven by mutations. This approach emphasises the importance of ecological context.

To fill the gap between the two approaches to games and investigate the ecological meaning of individual interactions we should answer the question: How do the outcomes of particular interactions affect the growth rates of the respective strategies? The methods related to game theory are also used in life history theory (Caswell 2001) to describe the competition between different life history strategies, but this framework does not assume interactions between individuals. In this approach fitness components are described as the vital rates (birth and death rates of the respective age or stage classes). We can use this approach to solve the posed problem and establish the link between interaction rates, describing the occurrence of the interaction events in time, and resulting vital rates of respective types of interactions, describing the changes of the population state. This question is important not only for game theoretic models. It is related to the problem of the general mathematical representation of fitness (Metz 2008; Roff 2008; Orr 2009) and the methodological interpretation of this term, discussed by biologists and philosophers of science (Mills and Beatty 1979; Rosenberg and Williams 1986; Horan 1994; Matthen and Ariew 2002; Brandon and Ramsey 2007; Matthen and Ariew 2009; Walsh 2010; Ramsey 2013).

### State of the art. An event-based approach

This paper extends a novel approach to evolutionary games from Argasinski and Broom (2012). This approach is focused on ecological realism, falsifiability and a mechanistic interpretation of the results obtained. The main goal was to express individual fitness in terms of demographic parameters. This allows us to describe the terms, such as “costs” and “benefits”, by measurable parameters (mortality interpreted as the probability of death and fecundity interpreted as the number of newborns obtained in effect through an interaction) instead of an abstract, undefined “fitness” described by an infinitesimal rate of increase of the population (or single component of fitness such as fecundity as in Chakra et al. (2014), where the number of eggs laid constitutes fitness). This is realized by the explicit application of two payoff functions describing mortality and fecundity counted in the currencies of births and deaths, instead of one fitness function describing excess from the mean Malthusian growth rate. This new approach can be described as event-based because it describes cause and effect chains of underlying interaction events. For example mortality can act on adult individuals before or after reproduction, or the description of the structure of the interaction event can be more complex.

In addition, this approach emphasises the role of density dependence. The fertility payoff functions are not constant in time but can be affected by selectively neutral juvenile mortality leading to a more complex selection mechanism induced by eco-evolutionary feedback (Hauert et al. 2006, 2008; Argasinski and Kozłowski 2008; Zhang and Hui 2011; Argasinski and Broom 2012; Huang et al. 2015; Gokhale and Hauert 2016). Thus the fertility reward can decrease, due to the increase of the juvenile mortality, below the adult mortality costs. Population size does not converge to an arbitrary phenomenological carrying capacity (constant, as in for example Cressman and Křivan 2010; Křivan 2013, or affected by payoffs, as in Novak et al. 2013) as in many models, exploiting the classical logistic growth, but to a dynamic equilibrium between all mortality and fertility factors. A similar approach that can be found in epidemiological models is called the emergent carrying capacity (Bowers et al. 2003; Sieber et al. 2014). This is more realistic and provides a mechanistic interpretation in terms of demographic factors. The properties of the selection mechanism, induced by strategically neutral growth limitation, were analysed in Argasinski and Broom (2013). Here at the population size equilibrium, newborns form a pool of candidates from which survivors which will replace dead adults at their nest sites will be drawn; this was termed the nest site lottery.

### Two research goals of the paper

**(a) Role of event occurrence rates describing the distribution of interaction events in time:** We will analyse how the rates of event (or interaction) occurrence, associated with respective event-related mortality and fertility payoffs, constitute the vital rates (rates of change of the population state, Caswell 2001) driving the population dynamics. However, the main difference between ecological population dynamics and evolutionary game theory is that population dynamics is focused on how the population is shaped by different types of events (which can be described by different types of games), while evolutionary game theory generally analyses the selection of strategies in a single particular type of event. Thus we should be able to extract those focal interaction from our more complex general model, with the remaining events generating the corresponding background fitness. The application of event occurrence rates (or interaction rates) for evolutionary games was originated in Taylor and Nowak (2006). In their paper different strategy carriers can interact at different rates and thus can play different numbers of game rounds (the differences with our approach, related to the definition of fitness, are discussed in section “The event-based approach and rates of event occurrence” in the Discussion.

**(b) Classification of the types of background fitness:** Traditionally, background fitness has been modelled by a phenomenological additive element of the payoff function that vanishes under the replicator dynamics, with the associated dynamics being very simple. The growth rate is described by a single payoff function and it is not clear whether background fitness is a neutral element of the payoff or a separate factor acting at a different occurrence rate. Within classical evolutionary game theory, which is density independent, both approaches are equivalent and distinction between them is not necessary. In addition there is no clear biological interpretation of this factor and it has rather been interpreted as a technical element of mathematical notation. As was shown in Argasinski and Broom (2012), background fitness components can seriously affect the dynamics. However, the natural interpretation of those factors can be provided by the approach from point a). Thus, can we derive phenomenological neutral elements as the aggregated outcomes of background events?

## Results

In the coming sections we will introduce a number of important terms used in our paper; a summary of these is presented in Table 1.

**Table 1:** A list of important symbols

Symbol	Description
*n*	Population size
$W^{i}$	Fertility payoff function of the *i*-th type event
$d^{i}$	Mortality payoff function of the *i*-th type event
*K*	Carrying capacity (maximal environmental load)
$q_{i}$	Frequency of the *i*-th strategy
$W_{i}(q)$	Fertility payoff of the *i*-th strategy
$s_{i}(q)$	Pre-reproductive survival payoff function of the *i*-th strategy
$V_{i}(q)$	Mortality-fertility trade-off function for the *i*-th strategy
$\tau _{i}$	Rate of occurrence (intensity) of the *i*-th type event
$\tau _{F}$	Rate of occurrence (intensity) of the focal game event
$\tau _{B}$	Rate of occurrence of the background event
$\tau$	Interaction rate from Argasinski and Broom (2012)—see “Appendix 1”
$\theta =\tau _{B}/\tau _{F}$	Average number of background events between two focal events
$W_{b}$	Focal game background fertility (payoff based approach)
$d_{b}=1-s_{b}$	focal game background post-reproductive mortality (payoff-based approach)
$W_{B}$	Average background event fertility (dynamics-based approach)
$d_{B}=1-s_{B}$	Average background event mortality (dynamics-based approach)
$\Phi =\theta W_{B}$	Rate of the average background fertility
$\Psi =\theta (1-s_{B})$	Rate of the average background mortality
*S*	Hawk–Dove example survival payoff matrix
$F=WP$	Hawk–Dove example fertility payoff matrix
$d=1-s$	Probability of death during a Hawk–Dove contest
$\tilde{q}_{h}(n)$	Frequency nullcline describing the Nash equilibria
$\tilde{n}(q_{h})$	Density nullcline describing the ecological equilibria

### The general model

#### Introduction of the rates of event occurrence and derivation of the vital rates

**Fig. 1:**
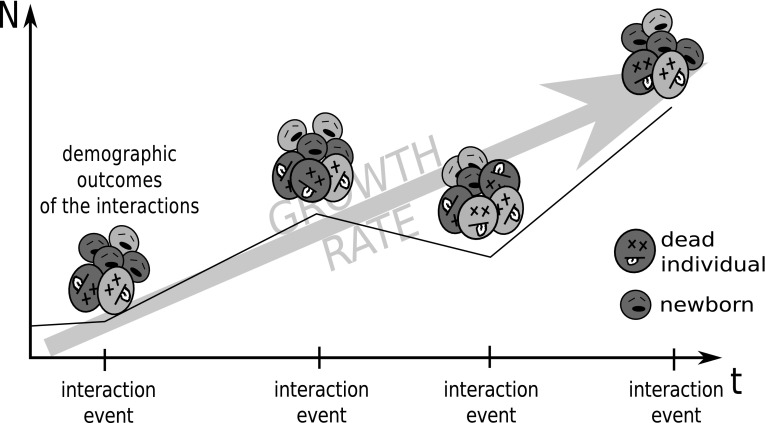
Schematic presentation of the idea underlying the proposed framework. Interaction events occur at some rate and the aggregation of their demographic outcomes (births and deaths) is responsible for changes of the population state

Firstly, let us derive the general growth equation according to the framework proposed in point (a) from “Two research goals of the paper”. We can consider multiple event types which occur as independent Poisson processes. Then, during a short time interval some number of events occur and their outcomes change the state of the population (newborns are introduced to the population and dead individuals are removed, see Fig. 1).

The events of the *i*-th type occur at rate $\tau ^{i}$ (the superscript describes the event type since later the subscript will describe the strategy), where its outcomes are described by respective fertility and mortality payoff functions $W^{i}$ and $d^{i}$, where $W^{i}$ is the average number of newborns produced and $d^{i}$ is the probability of death during this type of interaction event. If the *i*-th event type is a safe mating opportunity then the respective death probability $d^{i}$ equals zero. On the other hand, if the event is not related to mating or reproduction but is dangerous, then $W^{i}=0$. The general growth equation thus has the following form:

1 $$\begin{aligned} \dot{n}=\sum _{i}n\tau ^{i}\left( W^{i}-d^{i}\right) . \end{aligned}$$

Note that the parameters $\tau ^{i}$ do not have to be just technical constants but can be functions of the population size or strategic composition, as for example in the dynamic sex ratio model as we discuss in “The event-based approach and rates of event occurrence” in the Discussion (see Argasinski 2012, 2013, 2017) or models of upstream reciprocity (Nowak and Roch 2007; Pena et al. 2011). Thus we can derive the per capita vital rates as products of the event occurrence rates and the demographic outcomes of events. Then $\tau ^{i}W^{i}$ will be the fertility rate and $\tau ^{i}d^{i}$ will be the mortality rate for the *i*-th type of event. The sum of the respective vital rates over all types of events will constitute the crude mortality and fertility rates (Caswell 2001).

We will next apply the approach presented in this section and summarized by Eq. (1) to obtain the evolutionary dynamics framework centred on a particular focal game. We will extract one particular type of event from our general model to analyse the selection of individual strategies related to that game. The aggregated impact of all other types of events will constitute the background fitness.

#### Background fitness as the aggregated outcomes of background events

Individuals enter an arbitrarily chosen focal game (with payoffs $W_{F}$ and $d_{F}$ where auxiliary lower index *F* means “focal event”) at rate $\tau _{F}$ as in Eq. (1), and engage in other activities at rates described by $\tau _{B}^{i}$; we can consider a single class of all such activities, as we show below.

Each of the background events can be characterised by outcomes which include a fertility $W_{B}^{i}$ and mortality $d_{B}^{i}$ component (lower index *B* means “background event”). We can calculate the outcomes of the average background event. $W_{B}=\sum _{i}\tau _{B}^{i}W_{B}^{i}/\tau _{B}$ is the average fertility per event (where $\tau _{B}=\sum _{i}\tau _{B}^{i}$) and $d_{B}=\sum _{i}\tau _{B}^{i}d_{B}^{i}/\tau _{B}$ is the average death probability per event.

In effect “background events” occur at intensity $\tau _{B}$ and individuals involved in those events obtain fertility $W_{B}$ on average and survive with probability $s_{B}=1-d_{B}$. Then Eq. (1) can be presented in the following form:

2 $$\begin{aligned} \dot{n}=n\tau _{F}\left( W_{F}-d_{F}\right) +n\tau _{B}\left( W_{B}-d_{B}\right) . \end{aligned}$$

Note that, since the focal game depends on the analysed traits then the respective focal game payoffs will depend on the strategic composition of the population. However, for simplicity, the argument term (*q*) will not be included in the numbered equations. Then the interaction rates multiplied by demographic payoffs will constitute the “vital rates” (per capita rates of change of the population state, see Caswell 2001). Now we can extend our model to the detailed description of the evolutionary game including the different strategies. Each strategy should be represented by its respective equation of type (2) and assigned demographic payoff functions $$W_{F}(q)$$ and $$d_{F}(q)$$. We can use the structure of the demographic payoff functions from Argasinski and Broom (2012) (see “Appendix 1” for the necessary details) allowing us to describe the mortality and fertility outcomes of the elements of the causal chain underlying the single interaction event. We limit ourselves to the simple trade-off between a single pre-reproductive mortality stage and a single fertility stage (this happens for example in mating conflicts when males fight and the surviving winners can mate; thus mortality acts before reproduction). For each strategy $$W_{F}(q)\mathbf {\ }$$will be the mortality–fertility trade-off function $$V_{i}(q)= \sum _{j}q_{j}s_{i}(e_{j})W_{i}(e_{j})$$ describing the reproductive success of the survivors of the mortality stage described by survival payoff $$ s_{i}(q)=1-d_{i}(q)$$ (where index *i* describes the strategy number) acting as $$1-d_{F}(q)$$. In addition, we will include the density dependent juvenile survival function $$(1-n/K)$$ to introduce the nest site lottery mechanism (Argasinski and Broom 2013). Thus the general growth equation for the *i*-th strategy will be as follows:

3 $$\begin{aligned} \dot{n}_{i}=n_{i}\tau _{F}V_{i}\left( 1-\frac{n}{K}\right) -n_{i}\tau _{F}(1-s_{i})+n_{i}\tau _{B}W_{B}\left( 1-\frac{n}{K}\right) -n_{i}\tau _{B}d_{B}. \end{aligned}$$

We can adjust the timescale to make the focal game’s vital rates equal to their demographic payoffs. This will keep the mechanistic interpretation of the payoffs as the number of offspring and the survival probability during the interaction event. It is clear that only the ratio of our two interaction rates is important for the evolution of the population. After a change of timescale $\tilde{t}=t\tau _{F}$, $\tau _{F}$ vanishes and $\tau _{B}$ transforms into $\theta =\dfrac{\tau _{B}}{\tau _{F}}$. Note that letting $\tau _{F}$ tend to zero, i.e. letting $\theta$ tend to $\infty$, implies the weak selection limit where the impact of the focal game on the ecological dynamics vanishes; thus the eco-evolutionary feedback is broken (see “Formulation of a Hawk-Dove game as an example”. The parameter $\theta$ can be interpreted as the average number of background events between two focal interactions. Then the growth equation will be:

4 $$\begin{aligned} \dot{n}_{i}=n_{i}\left[ V_{i}\left( 1-\frac{n}{K}\right) -\left( 1-s_{i}\right) +\theta \left( W_{B}\left( 1-\frac{n}{K}\right) -d_{B}\right) \right] , \end{aligned}$$

leading to the following equation for the population size:

5 $$\begin{aligned} \dot{n}=\sum _{i}\dot{n}_{i}=n\left[ \sum _{i}q_{i}V_{i}\left( 1-\frac{n}{K} \right) -\left( 1-\sum _{i}q_{i}s_{i}\right) +\theta \left( W_{B}\left( 1-\frac{ n}{K}\right) -d_{B}\right) \right] . \end{aligned}$$

Parameters $\tau _{B}$, $W_{B}$ and $d_{B}$ can be biologically justified and can even be functions of other parameters (for example from other types of game). However, if we need only some background “noise” without particular justification, to add realism to our model, we can simplify the notation. Since demographic parameters $W_{B}$ and $d_{B}$ never occur without the ratio between intensities $\theta$, we can simplify this by substitutions $\Phi =\theta W_{B}$ and $\ \Psi =\theta d_{B}$, constituting the background vital rates. Letting $q_{i}=n_{i}/n$, we obtain the following system of replicator equations:

6 $$\begin{aligned} \dot{q}_{i}=q_{i}\left[ \left( V_{i}-\sum _{j}q_{j}V_{j}\right) \left( 1- \frac{n}{K}\right) +\left( s_{i}-\sum _{j}q_{j}s_{j}\right) \right] , \end{aligned}$$

7 $$\begin{aligned} \dot{n}=n\left[ \left( \Phi +\sum _{i}q_{i}V_{i}\right) \left( 1-\frac{n}{K} \right) +\sum _{i}q_{i}s_{i}-1-\Psi \right] , \end{aligned}$$

where Eq. (7) follows directly from Eq. (5), and Eq. (6) is obtained using Eqs. (4) and (5). The attractor of the population size is given by the nontrivial zero of the right-hand side of Eq. (7), constituting the density nullcline:

8 $$\begin{aligned} \tilde{n}=\left( 1-\dfrac{\Psi +1-\sum _{i}q_{i}s_{i}}{\Phi +\sum _{i}q_{i}V_{i}}\right) K. \end{aligned}$$

This approach to the background fitness can be termed the *dynamics based approach* since it is not related to the game theoretic structure. Note that this approach is related to the methodology used for the separation of ecological equations from selection dynamics (Cressman and Garay 2003a, b; Cressman et al. 2001). However, here we do not want to separate the ecological dynamics from the selection dynamics, since we believe that the relationship between ecology and selection is extremely important.

#### Two distinct approaches to background fitness

The background fitness vital rates, representing the impact of other games played by individuals, appear as the additive elements $\Phi \left( 1-\frac{n }{K}\right)$ and $\Psi$ in Eq. (7). However, traditionally in evolutionary games, a background fitness is represented by a background payoff which is the strategically neutral element of the payoff function (such as a constant added to all entries of the payoff matrix). But in our case, we have two separate payoffs described in distinct units (numbers of births and probability of survival). The question of whether the game theoretic background payoff concept and the background fitness describing the impact of the other games are equivalent arises. The impact on the dynamics of the neutral elements of both payoff functions is analysed in “Appendix 2”. It is shown there that only multiplicative pre-reproductive survival will be selectively neutral and will affect only the pace of convergence. Additive background fertility $W_{b}$ and multiplicative post-reproductive background survival $s_{b}$ (which was described by *m* in Argasinski and Broom 2012) will appear together in the multiplicative factor $\left( W_{b}\left( 1-\frac{n}{K}\right) +s_{b}\right)$ of the survival payoffs $s_{i}$ (this approach was used in Argasinski and Broom 2012). Note that here we use the lower case subscript *b* for the neutral elements of the payoff functions (that can be termed the *payoff-based approach*), to distinguish them from the payoffs from the alternative approach, where we use *B*. The replicator dynamics will be

9 $$\begin{aligned} \dot{q}_{i} & = q_{i}\left[ \left( V_{i}-\sum _{j}q_{j}V_{j}\right) \left( 1- \frac{n}{K}\right) +\left[ W_{b}\left( 1-\frac{n}{K}\right) +s_{b}\right] \left( s_{i}-\sum _{j}q_{j}s_{j}\right) \right] , \end{aligned}$$

10 $$\begin{aligned} \dot{n}& =  n\left[ \sum _{i}q_{i}V_{i}\left( 1-\frac{n}{K}\right) +\left[ W_{b}\left( 1-\frac{n}{K}\right) +s_{b}\right] \sum _{i}q_{i}s_{i}-1\right] , \end{aligned}$$

and then the manifold representing the population size equilibria (the *n* -nullcline, which is the attractor in the *n*-subspace) is

11 $$\begin{aligned} \tilde{n}=K\left( 1-\dfrac{1-s_{b}\sum _{i}q_{i}s_{i}}{W_{b} \sum _{i}q_{i}s_{i}+\sum _{i}q_{i}V_{i}}\right) . \end{aligned}$$

The above equations show that the neutral elements of the payoff functions produce different outcomes than the dynamics-based background fitness $\Phi$ and $\Psi$. However, the payoff-based approach can be a valuable element of the theoretical framework. In particular, it can be used to describe the selectively neutral elements linked with the game interaction such as juvenile mortality (responsible for the nest site lottery mechanism, Argasinski and Kozłowski 2008; Zhang and Hui 2011; Argasinski and Broom 2012, 2013). But it can be problematic, if we want to use it when describing the impact of other games, since it is an element of the causal chain of the focal game. This can be done only in the case of the background post-reproductive mortality $d_{b}=1-s_{b}$, which can be linked with background mortality $\Psi$ by the relationship described by Theorem 1 in “Appendix 2”.

The difference between the two approaches relies on the different distributions of events in time. In the dynamics-based background fitness $\Psi$ all background deaths gradually aggregate according to the intensities of all other games. In the payoff-based approach, all background deaths occur at the same time with the focal interaction as the last element of the causal chain (some survivors of the game are killed). Theorem 1 (see “Appendix 2”) shows that this mortality can be interpreted as the aggregated mortality between two focal game events.

If we limit analysis to the static case, then the interpretation of the background fitness as the mortality between two focal games is more natural and allows us to get rid of the instantaneous rates of occurrence from our reasoning. In addition this allows us to remove the abstract terminology of differential equations. In effect, the static reasoning can be expressed in clear, intuitive and empirically measurable terms, describing the respective causal stages of the interaction.

However, if we are interested in the dynamics, the differences related to the different distribution of deaths in time can seriously affect the predictions. This will be illustrated in the next section.

### Formulation of a Hawk–Dove game as an example

We will illustrate the results from “The general model” by use of a Hawk–Dove example. Argasinski and Broom (2012) considered the payoff matrices *S* (survival probability) and *P*, where the fertility matrix is $F=WP$, below: Here $s=1-d<1$ is the survival probability of a fight between Hawks, and the fertility matrix contains the expected number of newborns *W* produced from the interaction. This leads to the following set of replicator equations (see “Appendix 3” for a detailed derivation):

12 $$\begin{aligned} \dot{q}_{h}& =  q_{h}\left( 1-q_{h}\right) \left( \frac{1}{2}W\left( 1-q_{h}d\right) \left( 1-\dfrac{n}{K}\right) -q_{h}d\right) , \end{aligned}$$

13 $$\begin{aligned} \dot{n}& =  n\left( \left( \Phi +\frac{1}{2}W \left( 1-q_{h}^{2}d\right) \right) \left( 1-\frac{n}{K}\right) -q_{h}^{2}d-\Psi \right) , \end{aligned}$$

describes the Hawk frequencies and total population size. The zeros of the right-hand sides of the above equations will give nullclines constituting the equilibria of selection and ecological subsystems. Two rest points of this system are $q_{h}=0$ and 1. A nontrivial rest point, which becomes the attracting nullcline describing the manifold representing the strategic equilibria, is given by

14 $$\begin{aligned} \tilde{q}_{h}(n)=\dfrac{\frac{1}{2}W\left( 1-\dfrac{n}{K}\right) }{d\left( \frac{1}{2}W\left( 1-\dfrac{n}{K}\right) +1\right) }. \end{aligned}$$

There is a stable population size at either $\tilde{n}=0$ or at the positive restpoint, which is conditional on the Hawk strategy frequency (describing the nullcline constituting the population size equilibrium manifold parametrized by $q_{h}$),

15 $$\begin{aligned} \tilde{n}(q_{h})=K\left( 1-\dfrac{q_{h}^{2}d+\Psi }{\frac{1}{2}W\left( 1-q_{h}^{2}d\right) +\Phi }\right) . \end{aligned}$$

**Fig. 2:**
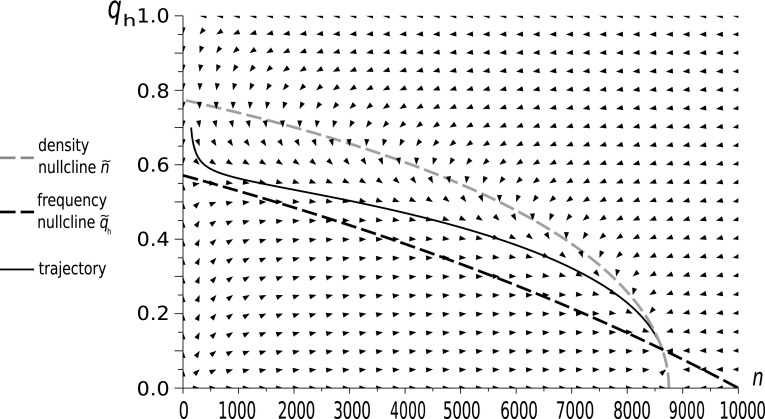
The dynamics of a Hawk–Dove population in our new model with initial conditions $q_{h}(0)=0.7$ and $n(0)=147$. Model parameters: $W=0.8$, $d=0.5$, $\Psi =0.006$, $\Phi =0.008$. In this case the impact of the background fitness components is very weak. The vector field indicated by the arrows shows that the force of attraction towards the density nullcline increases with population size. However, the dynamics does not converge quickly to the nullcline, and this case is far from timescale separation. Note that, the shape of the density nullcline highly depends on the strategic composition of the population. Thus the impact of the focal game on the population size is strong

**Fig. 3:**
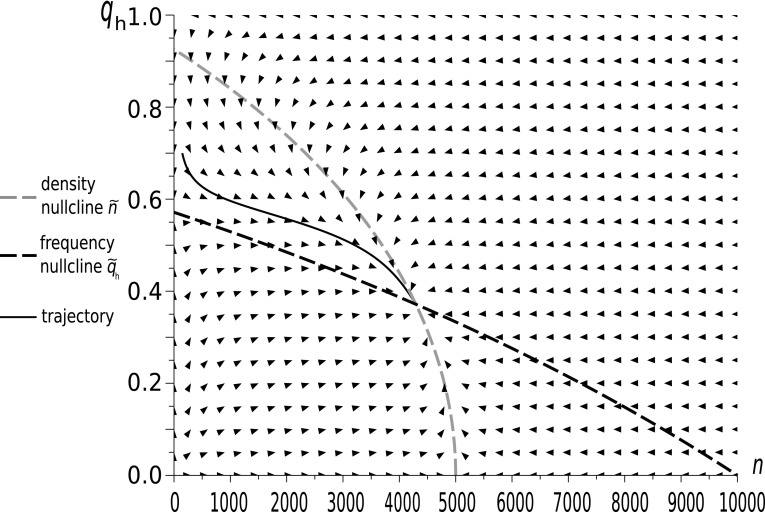
The dynamics of a Hawk–Dove population in our new model with initial conditions $q_{h}(0)=0.7$ and $n(0)=147$. Model parameters: $W=0.8$ , $d=0.5$, $\Psi =0.06$, $\Phi =0.08$. This case has background fitness components 10 times larger than in Fig. 1. The behaviour of the system and the restpoint have changed; however, the vector field depicted by the arrows shows that the system is still far from timescale separation

**Fig. 4:**
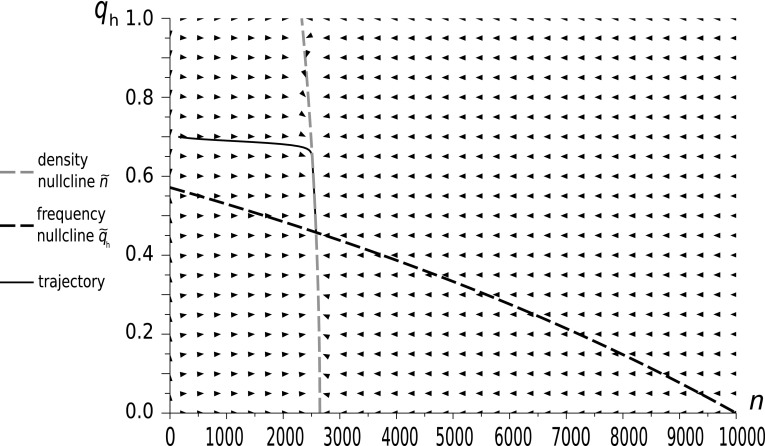
The dynamics of a Hawk–Dove population in our new model with initial conditions $q_{h}(0)=0.7$ and $n(0)=147$. Model parameters: $W=0.8$, $d=0.5$, $\Psi =15$, $\Phi =20$. In this case the impact of the background fitness components is very strong and the system is close to the weak selection limit. The stable restpoint is different to the restpoints from Figs. 2 and 3. Here the density nullcline is nearly flat due to the weak impact of the rare focal game events. The vector field described by the shows a strong attraction of the trajectory towards the density nullcline, then the trajectory traces it until it reaches the restpoint, i.e. we have effective timescale separation

Note that background fitness factors $\Psi \left( 1-\dfrac{n}{K}\right)$ and $\Phi$ affect the shape of the density nullcline. Numerical simulations show that this impact can be significant. This is illustrated by examples in Figs. 2, 3, 4, which differ only by parameters $\Phi$ and $\Psi$.

We see that when $\Phi$ and $\Psi$ are small as in Fig. 2, and to a lesser extent in Fig. 3, the trajectory is clearly distinct from the density nullcline $\tilde{n}(q)$, whereas for large $\Phi$ and $\Psi$, as in Fig. 4, the trajectory converges quickly to this nullcline, and then follows it to the equilibrium point. Thus, only for large $\Phi$ and $\Psi$ , i.e. in the weak selection limit, can we obtain a separation of timescales for slow frequency and fast size dynamics, which can be described by its equilibrium value.

The shape of the density nullcline shows the strength of the impact of the focal game, via eco-evolutionary feedback, on the ecology of the population. In Figs. 2 and 3, where the focal game is quite a frequent event (since background fitness is relatively low), the density nullcline (as a function of $q_{h}$) depends strongly on the strategic composition. On the other hand, in the case when the focal game is rare (as in Fig. 4), and so its impact is weak, the density nullcline is nearly flat.

#### Impact of the distribution of interaction events in time on the dynamics

In this section we will illustrate the relationships between the dynamics-based and payoff-based approaches to the background mortality analysed in “Two distinct approaches to background fitness” and summarized by Theorem 1 in “Appendix 2”. This will show the importance of the impact of the distribution of events in time. In the payoff-based approach, background deaths occur simultaneously with the focal game event while in the dynamics-based approach they gradually aggregate independently of the focal interactions. We can observe this comparing the numerical simulations of the system (12, 13) with the system (55, 56), which is the Hawk–Dove game model derived according to (9, 10). For simplicity we remove the background fertility from both systems by setting $\Psi$ and $W_{b}$ equal to zero.

**Fig. 5:**
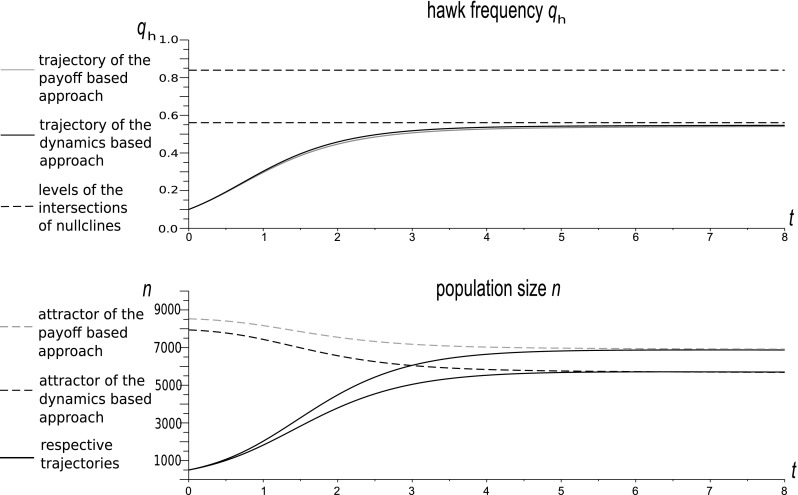
Time evolution of the Hawk frequency and population size for the payoff-based and dynamics-based models for the parameters: $$W=4$$, $$d=0.85 $$, $$\Psi =0.4$$. Levels of intersections indicate the frequency coordinates of the intersections of frequency and density nullclines $$\tilde{q}_{h}(n)$$ and $$\tilde{n}(q_{h})$$, constituting the rest points of the eco-evolutionary dynamics. The frequency predictions are similar but the trajectories of the population sizes differ significantly

**Fig. 6:**
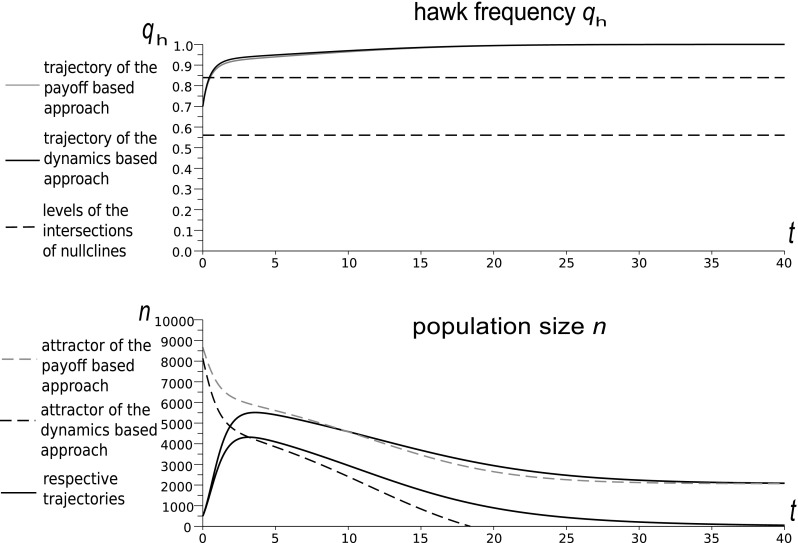
Time evolution of the Hawk frequency and population size for the payoff-based and dynamics-based models for the parameters: $W=15$, $d=0.85$, $\Psi =0.4$. As in Fig. 5, the frequency trajectories are similar but the predicted population sizes differ dramatically. The payoff-based model predicts a positive population size at the upper intersection. For the dynamics-based approach, the intersection describing the Hawk invasion barrier is in the region of extinction, since the stable population size is negative

**Fig. 7:**
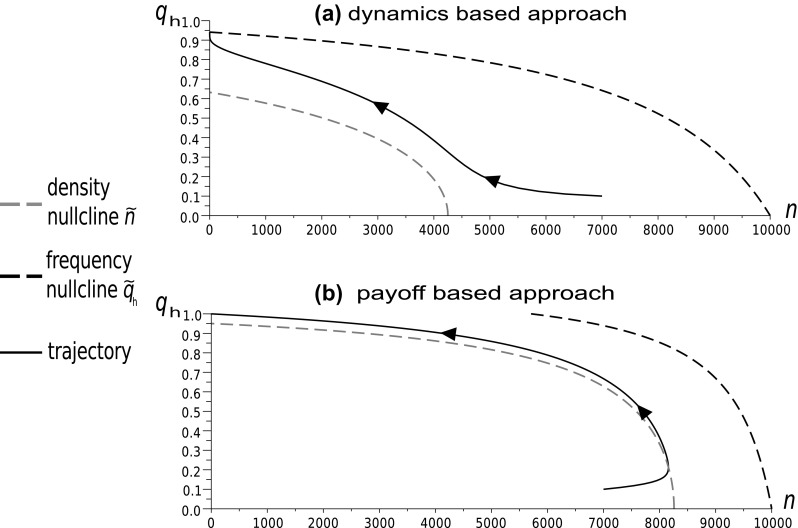
Phase diagrams following the evolution of the Hawk frequency and population size for the payoff-based and dynamics-based models for the parameters: $W=8$, $d=0.85$, $\Psi =2.3$. The frequency and density nullclines occupy different positions with respect to the same initial point in both cases, and the trajectories obtained are totally different

Numerical simulations show that for small background mortalities the two approaches produce similar trajectories of strategy frequencies, but ecological predictions differ significantly (see Figs. 5 and 6). The dynamics-based model can predict extinction in the case when the payoff-based model shows a positive stable population size (Fig. 6). These numerical results support the analytical results from Theorem 1 in “Appendix 2”, which shows that the stable sizes predicted by the model of Argasinski and Broom (2012) are biased, while the frequency levels of the intersections are the same for both approaches. With an increase of the background mortality, the differences between the models also increase and can affect frequency trajectories and phase portraits (Fig. 7). Thus the above example supports the claims that background fitness and the background payoff are distinct, although related, concepts.

## Discussion

### Two types of background fitness

Background fitness is traditionally interpreted as some phenomenological constant (or function) added to the payoffs of all strategies which vanishes from the continuous replicator equations. This concept can be found in many papers (for example see Cressman et al. 1986; Houston and McNamara 1991; Claussen and Traulsen 2005), but it is treated as a technical element of the mathematical notation and these works are not primarily focused on the biological meaning of it. Background fitness can be interpreted in two ways: First, as an element of the game theoretic structure (a generalization of the background payoff from classical game theory). Second, as an element of the dynamics occurring independently from the focal game at a separate rate of occurrence. In the basic approach to evolutionary game theory, the two approaches are indistinguishable. Some researchers, for instance those working explicitly using discrete dynamics or weak selection models, interpret the classical background fitness in rather a similar way to the first approach (Broom and Rychtar 2013; Taylor and Nowak 2006; Wu et al. 2010), while for others, for instance in optimal foraging/ diet choices models, the underlying logic involves the second approach (Křivan 1998, 2003; Cressman and Křivan 2010). However there was no rigorous formalization of this aspect and it is rather an example of “folk” knowledge.

Our work shows that there are essentially two types of background fitness (or more precisely, of background mortality and fertility), the payoff-based and the dynamics-based approaches. The dynamics-based approach that we focus on in this paper acts as the classical background fitness and is derived from the general ecological model, not phenomenologically postulated. While the payoff-based approach is a good tool to describe the selectively neutral factors related to the game interaction, which is clearly shown by the example of density dependent juvenile mortality (Argasinski and Kozłowski 2008; Zhang and Hui 2011; Argasinski and Broom 2012, 2013), we have shown here that such an application to the factors not related to the focal game can be problematic. The payoff-based approach does not take into account the distribution of the background events in time. The outcomes of all background events which occurred between two focal events affect the population state simultaneously when a single focal event occurs, since they are the final element of the focal game’s causal chain. The dynamics-based background fitness is free from this disadvantage. Note that both types of background fitness are not selectively neutral and affect the dynamics of the system via strategically neutral juvenile mortality (as is shown by a Hawk-Dove example). This impact is nontrivial and will probably affect the general stability conditions. This is a question which is analysed in a subsequent paper (Argasinski and Broom 2017).

### The event-based approach and rates of event occurrence

The most general and important claim resulting from our approach is that the payoff is not equivalent to the population growth rate. Game-theoretic notions describe the causal structure and the resulting outcomes of the specific single interaction. The interactions aggregate with some rate and the product of this rate with demographic outcomes constitute the vital rates. This is different to the approach from Taylor and Nowak (2006), where the fitness is expressed as the outcome of the average interaction (the sum of payoffs from interactions divided by the number of those interactions). This assumption does not take into account the impact of the different numbers of interactions on the fitness of the particular strategy. We can imagine an example when one strategy will obtain on average lower reproductive success per interaction than a competitor, but will participate in more interactions, so that its aggregated reproductive success is larger. The approach proposed here explicitly takes this into account. We thus believe that the number of games played should be explicitly considered. Then the demographic payoffs will describe the outcomes of the average interaction event (similarly to the stoichiometric coefficients in chemical kinetics, Upadhyay 2006) not the growth rates as in traditional evolutionary games.

In static models there is no time but causal consequences of the strategic “decisions” of individuals described by their reproductive success. Thus, the rigorous derivation of the population growth rate as an aggregated outcome of individual interactions needs rates of occurrence as the necessary element to make the framework consistent (the problem of the consistency and realism of modelling frameworks was discussed in Houston and McNamara 2005; McNamara 2013). Traditionally, interaction rates are not explicitly analysed in game-theoretic selection models (some exceptions will be discussed later). However, they can be a practical analytic tool. We can imagine a population of individuals where the type of games played depends upon their situation. Then the probabilities of finding particular situations associated with the respective type of game can be described by different rates of occurrence. The new approach corrects intuitions inspired by classical birth and death processes (see e.g. Haigh 2002) where birth and death events are described by different intensities, which implies statistical independence of births and deaths. In this case trade-offs between mortality and fertility are impossible. In evolutionary theory benefit is linked to reproductive success while expected cost is related to the associated mortality risk. The approach from this paper can be used to describe the correlations between mortality and fertility factors associated with particular activities.

In addition, rates of occurrence are not necessarily constants. For example they can be functions of the population size (more individuals implies potentially more interactions per unit time) or population state. A good example of this is the battle of the sexes with the problem of pair formation (Mylius 1999) or dynamic model of sex ratio evolution (see Argasinski 2012, 2013 and 2017). There an elementary event (a Bernoulli trial) is the production of a single offspring with a randomly chosen partner. Then females interact at a constant rate, while males interact proportionally to the number of available females described by the actual sex ratio (female to male). Thus interaction rates constitute a crucial element of the strategy selection mechanism. We can also imagine the situation when interaction rates can depend on an individual’s strategy. This can be illustrated by the example of non-uniform interaction rates in the models of social dilemmas, such as the models of upstream reciprocity (Nowak and Roch 2007; Pena et al. 2011). In this case there are different interaction rates for different individual strategies within the single game.

There is also an interesting relationship between the so-called weak selection concept and our eco-evolutionary feedback. Traditionally, in population genetics and models based on continuous traits, the weak selection limit assumes very small differences between strategic agents or alleles resulting in small selective advantage (Kimura 1968; Ohta 2002). This assumption was also used in matrix game models, where assumption of small differences is not necessarily applicable (for example in models with contrasting strategies such as Hawks and Doves which by definition will obtain different demographic payoffs). Then, the weak selection limit is introduced via a selection constant (see Wild and Traulsen 2007 for the comparison of both approaches). Note that the selection constant (Nowak et al. 2004; Antal et al. 2009; Taylor et al. 2004; Ohtsuki et al. 2006; Taylor et al. 2007; Fu et al. 2009; Tarnita et al. 2009a; Wild and Traulsen 2007) can be interpreted as the rate of the focal game’s occurrence. Under weak selection, a focal game described by relatively high demographic parameters will be a rare event, and its impact on the population dynamics will be small. Our interpretation embeds this concept in a clear biological context. The weak selection limit can be applied only in the case that the focal events are really rare. Thus it cannot be applied in common types of interactions such as mating conflicts or resource conflicts during foraging. Note that, in the weak selection limit, where our parameter $\theta$ tends to infinity, the impact of the frequency dynamics on the population size vanishes, but the second element of the eco-evolutionary feedback is still present. The frequency dynamics is affected by population size via juvenile survival inducing the nest site lottery mechanism on the density nullcline (Argasinski and Broom 2013).

### General discussion

The event-based approach constitutes a clearly defined area of application of game-theoretic notions within the evolutionary dynamics framework. The mathematical structure describing the focal interaction can be very complex (e.g. see Broom and Ruxton 1998; Gokhale and Traulsen 2010; Broom 2002; Broom and Cannings 2002, and in general the book Broom and Rychtar 2013) and a clear methodology how to incorporate the game into a population dynamic model can be important. The approach proposed in this paper shows that evolutionary dynamics under growth, limited by nest site availability, is a synergistic product of different games played by individuals, not only a simple aggregated sum of the outcomes of those games. Thus, the dynamical approach is more than an extension of the static game structure, as in classical theory. An important aspect of this approach is that it can be easily interpreted, which is a significant advantage over abstract simplified models , as argued Geritz and Kisdi (2012). Further, the proposed approach allows for more precise modelling of the outcomes of selection dynamics on ecological parameters such as population size. This is very important, because the relationship between ecological mechanisms (regulation of the population size) and the process of natural selection is one of the major problems of modern evolutionary biology (Birch 1960; Hutchinson 1965; Ginzburg 1983) and is still at the centre of debate (Pelletier et al. 2009; Morris 2011; Post and Palkovacs 2009; Schoener 2011). We note that aspects discussed above are important from the point of view of the general definition of fitness (see Metz 2008; Roff 2008; Orr 2009) and its interpretation within evolutionary theory (Mills and Beaty 1979; Rosenberg and Williams 1986; Horan 1994; Matthen and Ariew 2002; Brandon and Ramsey 2007; Matthen and Ariew 2009; Walsh 2010; Ramsey 2013).

The proposed approach shows how the game theoretic notions, causal structure underlying the interactions that shape the population dynamics, can be used within many theoretical frameworks. It can be easily extended to Adaptive Dynamics (Dercole and Rinaldi 2008) due to its clear description of the underlying ecology. On the other hand, decomposition of fitness into separate demographic payoffs creates the possibility of incorporating more detailed population genetic mechanisms (Crow and Kimura 1970; Hartl and Clark 1997; Bürger 2000), which will affect the fertility payoffs. Then the fertility payoff will describe the number of mating attempts which can be weighted by the probability of gene transfer determined by the underlying genetic system.

In addition the impact of the proposed methodology can be broader and more general. Note that in the case of abstract model parameters, that are “fitted” to data, it can be hard to falsify the obtained outcomes, if the model is “flexible” enough with respect to the “fitted” parameters, to cover different types of datasets. The clear demographic meaning of our model parameters can allow for easy falsification according to empirical data or the outcomes of individual-based simulations (which will have the status of in-silico experiments, see Uchmański and Grimm 1996; Grimm and Railsback 2005) parameterized by the same values. The predictions of the analytical model can be helpful in a mechanistic explanation of the patterns produced by the simulation (see, for example, Gerlee and Lundh 2010) or observed empirical data. This will constitute important progression in the direction of the development of theoretical notions related to the individual level, originated by Łomnicki (1988), and in particular related to research on animal personalities (Dall et al. 2004; Wolf et al. 2007; Wolf and Weissing 2010, 2012; Wolf and McNamara 2012). Thus, the event-based terminology not only extends the mathematical notions, but also influences the general theory and contributes to the understanding of the causal structure of the evolutionary process.

## Appendix 1: The details of the general model from Argasinski and Broom (2012)

We now briefly consider some important ideas and terminology from Argasinski and Broom (2012). The main idea underlying the modelling approach of Argasinski and Broom (2012) is that all interactions between individuals occur with single intensity $\tau$ (there are no distinction between different games played by the individuals) and their outcomes are described by demographic payoff functions, fertility *W* (interpreted as the number of offspring produced during an interaction) and mortality *d* (interpreted as the probability of death during an interaction). This leads the following growth equation:

16 $$\begin{aligned} \dot{n}_{i}=n_{i}\tau \left( W_{i}-d_{i}\right) . \end{aligned}$$

The paper was mostly focused on the description of the causal stages of the game interaction. In this paper we will not analyse this aspect; thus for the objectives of the current paper, we do not need a complex causal structure for our focal interaction. We will limit ourselves to a single pre-reproductive mortality stage. Assume that it is described for the *i*-th strategy by payoff functions $$ s_{i}$$ and single fertility stage (when survivors reproduce) described by payoff functions $$W_{i}$$. For our population, $$q_{i}=n_{i}/n$$ is the frequency of strategy *i*, $$n=\sum n_{i}$$ the population size, *K* the carrying capacity (maximum environmental load, Hui 2006). Since we assumed the pre-reproductive survival stage, which implies that only survivors can reproduce, this leads to a mortality–fertility tradeoff. Since $$ s_{i}$$ and $$W_{i}$$ will be functions of vector *q* (frequency dependent, as in Framework II in Argasinski and Broom 2012) the mortality–fertility trade-off function $$V_{i}(q)=\sum _{j}q_{j}s_{i}(e_{j})W_{i}(e_{j})$$ should be introduced. Finally, interactions occur with rate of occurrence $$\tau $$. The population for each individual strategy growth equation can be formulated as follows:

17 $$\begin{aligned} \dot{n}_{i} & = n_{i}\tau V_{i}\left( 1-\frac{n}{K}\right) -n_{i}\tau (1-s_{i}) \end{aligned}$$

18 $$\begin{aligned}& = n_{i}\tau \left( V_{i}\left( 1-\frac{n}{K}\right) -\left( 1-s_{i}\right) \right) . \end{aligned}$$

The above equation is the extended version of (16) where $$W_{i}(q)$$ is replaced by $$\left( 1-\frac{n}{K}\right) V_{i}(q)$$ and $$d_{i}(q)=(1-s_{i}(q))$$ are game theoretic fertility and mortality payoffs. This introduces the simple trade-off between mortality and fertility. In the Eq. (17) we can remove the interaction rate $$\tau $$ by a change of timescale (see “Appendix 1” in Argasinski and Broom 2012). Then, the above system can be rescaled to the related frequencies. These assumptions lead to the following detailed general system of replicator equations (describing the changes of strategy frequencies $$ q_{i}$$) including an equation on population size *n*, which will be analysed in this paper:

19 $$\begin{aligned} \dot{q}_{i}& =  q_{i}\left[ \left( V_{i}-\sum _{j}q_{j}V_{j}\right) \left( 1- \frac{n}{K}\right) +\left( s_{i}-\sum _{j}q_{j}s_{j}\right) \right] , \end{aligned}$$

20 $$\begin{aligned} \dot{n}& =  n\left( \sum _{i}q_{i}V_{i}\left( 1-\frac{n}{K}\right) +\sum _{i}q_{i}s_{i}-1\right) . \end{aligned}$$

From the Eq. (20) we can derive the description of the *n*-nullcline (which is the attractor in *n*-subspace):

21 $$\begin{aligned} \tilde{n}=K\left( 1-\dfrac{1-\sum _{i}q_{i}s_{i}}{\sum _{i}q_{i}V_{i}}\right) . \end{aligned}$$

This nullcline consists of the equilibria of the ecological dynamics.

## Appendix 2: A comparison between the new (dynamics-based) approach and the approach of Argasinski and Broom (2012)

The background fitness terms derived in the “Background fitness as the aggregated outcomes of background events” (dynamics-based approach) act as additive factors that vanish from the replicator dynamics (in a similar way as in the classical theory). However, in the classical game theory, the background payoff is the neutral (additive) element of the payoff function. Thus it is an element of the game theoretic structure. Are background vital rates $$\Phi $$ and $$\Psi $$ equivalent to the neutral elements of the demographic payoff functions $$W_{i}$$ and $$s_{i}$$? This can be shown by comparison of the approach (which we call here the payoff-based approach) used in Argasinski and Broom (2012), where all interaction events occur at the single intensity $$\tau $$ (there are no distinctions between different game types) and the background fitness components are described by phenomenological, strategically neutral elements of the payoff functions. Neutral pre-reproductive mortality will be represented by a multiplicative factor of the whole right-hand side of replicator and population size equations (since it will be the first element of the interactions causal chain); thus it will affect only the pace of convergence. Therefore, it can be incorporated into the occurrence rate of the focal game $$\tau _{F}$$ as an adjustment. The situation is more complicated with the additive element $$ W_{b}+W_{i}$$ in the fertility payoff and multiplicative post-reproductive mortality $$d_{b}=1-s_{b}$$ affecting the survivors of the game mortality stage $$s_{i}d_{b}$$). This will lead to the growth equation

22 $$\begin{aligned} \dot{n}_{i}& =  n_{i}\tau \left( V_{i} \left( 1- \frac{n}{K}\right) -\left( 1-s_{i}\right) +s_{i}W_{b}\left( 1-\frac{n}{K} \right) -s_{i}d_{b}\right) \nonumber \\& =  n_{i}\tau \left( V_{i}\left( 1-\frac{n}{K}\right) -1+s_{i}\left[ W_{b}\left( 1-\frac{n}{K}\right) +1-d_{b}\right] \right) . \end{aligned}$$

Thus the selectively neutral elements appear as the multiplicative factor $\left[ W_{b}\left( 1-\frac{n}{K}\right) +1-d_{b}\right]$ of the survival payoffs $s_{i}$. This leads to the following extension of the system (19, 20):

23 $$\begin{aligned} \dot{q}_{i}& =  q_{i}\left[ \left( V_{i}-\sum _{j}q_{j}V_{j}\right) \left( 1-\frac{n }{K}\right) +\left[ W_{b}\left( 1-\frac{n}{K}\right) +s_{b}\right] \left( s_{i}-\sum _{j}q_{j}s_{j}\right) \right] , \end{aligned}$$

24 $$\begin{aligned} \dot{n}& =  n\left( \sum _{i}q_{i}V_{i}\left( 1-\frac{n}{K}\right) +\left[ W_{b}\left( 1-\frac{n}{K}\right) +s_{b}\right] \sum _{i}q_{i}s_{i}-1\right) . \end{aligned}$$

Then the density nullcline will be:

25 $$\begin{aligned} \tilde{n}=\left( 1-\dfrac{1-s_{b}\sum _{i}q_{i}s_{i}}{W_{b} \sum _{i}q_{i}s_{i}+\sum _{i}q_{i}V_{i}}\right) K. \end{aligned}$$

This is system (9, 10). Thus the dynamics-based approach from “Background fitness as the aggregated outcomes of background events”, based on parameters $\Phi$ and $\Psi$, is somewhat simpler than the original equations from Argasinski and Broom (2012), (23) and (24). In addition, it is clear that there is no need for a distinction between pre- and post-reproductive mortality, because those factors are totally independent of the focal game. Thus the classical approach to the background fitness should be interpreted as the aggregated outcome of other events (games) occurring independently to the focal game, not as a phenomenological element of the payoff function of the focal game itself.

A question arises about the relationship between the two approaches. First, let us compare the approaches with respect to the background fertility. In the payoff-based approach (Argasinski and Broom 2012) this factor is represented by $$\left( 1-\frac{n}{K}\right) W_{b}(s_{i}-\sum _{j}q_{j}s_{j})$$ in the frequency equation (23) and by $$\left( 1-\frac{n}{K}\right) W_{b}\sum _{i}q_{i}s_{i}$$ in the population size equation (24). $$W_{b} $$ is multiplied by the averaged pre-reproductive mortality related to the focal game, since it describes a strategically neutral element of the fertility stage of the focal game’s causal chain. In the dynamics-based approach it is represented by $$\left( 1-\frac{n}{K}\right) \Phi $$ in the population size equation (7) only. In the dynamics-based approach the whole factor is represented by a single constant $$\Phi $$ and is independent of the focal game. Thus the above two approaches produce clearly distinct results.

The dynamics-based approach seems to be more natural for the modelling of the impact of other interaction events which occur other than through the focal game. In addition it is technically equivalent to the approach used in classical theory. This suggests that the background fitness is not the same as the background payoff in classical game theory leading to the payoff-based approach from Argasinski and Broom (2012). Thus, both approaches are not equivalent with respect to the cases described above. However, in the case of post-reproductive mortality some relationship with background mortality $\Psi$ can be shown.

Let us compare the general size equations using the different approaches to the background mortality, where $s_{b}$ is the neutral post-reproductive survival of the payoff-based approach used in Argasinski and Broom (2012) and $s_{B}$ is the dynamics-based approach from “Background fitness as the aggregated outcomes of background events”. For the dynamics-based approach we have

26 $$\begin{aligned} \dot{n}_{i}=n_{i}V_{i}\left( 1-\frac{n}{K}\right) -n_{i}(1-s_{i})-n_{i}\Psi , \end{aligned}$$

whereas the equation for the payoff-based approach was

27 $$\begin{aligned} \dot{n}_{i}=n_{i}V_{i}\left( 1-\frac{n}{K}\right) -n_{i}(1-s_{i})-n_{i}s_{i}d_{b}. \end{aligned}$$

Thus the decay rates constituted by the mortality terms in equations (26) and (27) are

28 $$\begin{aligned} E_{1}=-(1-s_{i})-\Psi \end{aligned}$$

for the dynamics-based approach, and

29 $$\begin{aligned} E_{2}=-(1-s_{i})-s_{i}d_{b} \end{aligned}$$

for the payoff based approach. Note that equality of the decay rates of both approaches $E_{1}=E_{2}$ implies the condition

30 $$\begin{aligned} s_{i}d_{b}=\theta d_{B} \end{aligned}$$

which cannot be satisfied for every *i*; thus they are not equivalent. Now, let us focus on the relationship between the two approaches (distinguished by the indexes *dyn* for dynamics-based approach and *pf* for the payoff-based approach). We can use a Poisson process theory (Haigh 2002), to show conditions when the payoff-based approach can be treated as an approximation of the dynamics-based approach:

### **Theorem 1**

*Assuming that* $s_{b}=\dfrac{1}{1+\Psi }$ *and the population size equilibrium condition* (8) *is satisfied, then*

- *The stationary frequency points for both approaches are the same.*
- *The rate of the dynamics in the payoff-based approach equals the* *rate of the dynamics-based approach divided by* $1+\Psi$ (*or alternatively multiplied by* $s_{b}$).
- *Juvenile survival probabilities* $J_{\mathrm{dyn}}=\left( 1-\frac{\tilde{n}_{\mathrm{dyn}}}{K }\right)$ *and* $J_{pf}=\left( 1-\frac{\tilde{n}_{pf}}{K}\right)$ *at the stable state and respective stable population sizes* $\tilde{n}_{\mathrm{dyn}}$ *and* $\tilde{n}_{pf}$ *are different and* $J_{\mathrm{dyn}}=J_{pf}/s_{b}$ *leading to*
  31 $$\begin{aligned} \tilde{n}_{\mathrm{dyn}}=(1-J_{pf}/s_{b})K=\left( 1-\dfrac{1-s_{b}\sum _{i}q_{i}s_{i}}{ s_{b}\sum _{i}q_{i}V_{i}}\right) K. \end{aligned}$$

We give a proof below.

Thus, if we are only interested in the static analysis, it is possible to approximate independent background mortality by post-reproductive mortality $d_{b}=1-s_{b}=\dfrac{\Psi }{1+\Psi }$ related to the game event, describing the aggregated mortality caused by independent background events occurring between two game interactions. Stable frequencies will be the same and the respective juvenile survivals and population sizes can be found according to part c) of Theorem 1.

### *Proof of Theorem 1*

Parts (a) and (b). The replicator equations from Argasinski and Broom (2012) using the payoff-based approach to post-reproductive background mortality (a special case of (23) with $W_{b}=0$) are given by

32 $$\begin{aligned} \dot{q}_{i}=q_{i}\left[ \left( V_{i}-\sum _{j}q_{j}V_{j}\right) \left( 1- \frac{n}{K}\right) +s_{b}\left( s_{i}-\sum _{j}q_{j}s_{j}\right) \right] , \end{aligned}$$

and for the density nullcline we have the following form of juvenile survival:

33 $$\begin{aligned} 1-\frac{\tilde{n}}{K}=\dfrac{1-s_{b}\sum _{i}q_{i}s_{i}}{\sum _{i}q_{i}V_{i}}. \end{aligned}$$

Using the basic properties of the Poisson process, for two types of events happening at rates $\nu _{1}$ and $\nu _{2}$, the probability that the first type event comes before the second type is $s_{b}=\dfrac{\nu _{1}}{\nu _{1}+\nu _{2}}$. In our case the game interaction happens at rate 1 (after the change of timescale $\tilde{t}=t\tau _{F}$) and background mortality acts at rate $\Psi =\theta (1-s_{B})$ (where $\theta =\tau _{B}/\tau _{F}$). Then the survival probability between game interactions is given by $s_{b}=\dfrac{1}{1+\Psi }$ since survival occurs if a new contest comes before death. It is easy to show that this relationship does not hold for the payoff-based approach in general. However, under the assumption of population size equilibrium, some relationships between the payoff-based approach as an approximation of the dynamics-based approach to the background mortality can be derived. After substitution of the population size equilibrium (33) into (32), we have

34 $$\begin{aligned} \dot{q}_{i}=q_{i}\left[ \left( V_{i}-\sum _{j}q_{j}V_{j}\right) \dfrac{1-s_{b}\sum _{i}q_{i}s_{i}}{\sum _{i}q_{i}V_{i}}+s_{b}\left( s_{i}-\sum _{j}q_{j}s_{j}\right) \right] . \end{aligned}$$

After substitution of $s_{b}=\dfrac{1}{1+\Psi }$ to the above equation, we have

35 $$\begin{aligned} \dot{q}_{i}=\dfrac{q_{i}}{1+\Psi }\left[ \left( V_{i}-\sum _{j}q_{j}V_{j}\right) \dfrac{\Psi +1-\sum _{i}q_{i}s_{i}}{ \sum _{i}q_{i}V_{i}}+\left( s_{i}-\sum _{j}q_{j}s_{j}\right) \right] . \end{aligned}$$

The right-hand side of Eq. (35) equals a constant times the r.h.s of Eq. (6) using the substitution from Eq. (8) (recall that we assumed $\Phi =0$ for simplicity). Thus $s_{b}$ only affects the rate of convergence and intersections of the nullclines will be the same as in the new method presented in this paper.

Part (c). Substituting the population sizes to the logistic suppression coefficient we can calculate the juvenile survivals on the density nullcline. Then

36 $$\begin{aligned} J_{pf}=\left( 1-\frac{n_{pf}}{K}\right) =\dfrac{1-s_{b}\sum _{i}q_{i}s_{i}}{ \sum _{i}q_{i}V_{i}} \end{aligned}$$

for the payoff based approach, and

37 $$\begin{aligned} J_{\mathrm{dyn}}=\left( 1-\frac{n_{\mathrm{dyn}}}{K}\right) =\dfrac{1-\sum _{i}q_{i}s_{i}+\Psi }{\sum _{i}q_{i}V_{i}}=\dfrac{1/s_{b}-\sum _{i}q_{i}s_{i}}{\sum _{i}q_{i}V_{i}} \end{aligned}$$

(since $\Psi =1/s_{b}-1)$, for the dynamics-based approach.

Since we are especially interested in the result of the dynamics-based approach and treat the payoff-based model as an approximation of the dynamics based one, we need to express the predictions of the dynamics-based model in terms of the payoff-based approach. Thus for a given set of parameters common to both models, we obtain from the above that

38 $$\begin{aligned} J_{pf}=s_{b}J_{\mathrm{dyn}} \end{aligned}$$

and the stationary population size for the dynamics-based model can be described as

39 $$\begin{aligned} \tilde{n}_{\mathrm{dyn}}=\left( 1-{\frac{J_{\mathrm{pf}}}{s_{b}}}\right) K. \end{aligned}$$

leading to (31). $$\square $$

## Appendix 3: Derivation of the Hawk–Dove example game

The equations using the Hawk–Dove payoff functions equivalent to the Eqs. (19) and (20), where fertility payoffs are $V_{i}=e_{i}S\cdot Pq^{T}W$ and $\sum _{j}q_{j}V_{j}=qS\cdot Pq^{T}W$ while survival payoffs are $s_{i}=e_{i}Sq^{T}$ and $\sum _{j}q_{j}s_{j}=qSq^{T}$ (where “$\cdot$” means elementwise multiplication and $e_{i}$ is the base vector with 1 on *i*-th coordinate and zeros on the others), will be the following:

40 $$\begin{aligned} \dot{q}_{h} =  q_{h}\left( W\left( e_{1}S\cdot Pq^{T}-qS\cdot Pq^{T}\right) \left( 1-\dfrac{n}{K}\right) +(e_{1}Sq^{T}-qSq^{T})\right) \end{aligned}$$

41 $$\begin{aligned} \dot{n}& =  n\left( qS\cdot Pq^{T}W\left( 1-\frac{n}{K}\right) +qSq^{T}-1\right) . \end{aligned}$$

The matrix operations are as follows:

42 $$\begin{aligned} e_{1}Sq^{T} =  sq_{h}+1-q_{h}=q_{h}(s-1)+1, \end{aligned}$$

43 $$\begin{aligned} e_{1}S\cdot Pq^{T} =  \frac{1}{2}sq_{h}+1-q_{h}, \end{aligned}$$

44 $$\begin{aligned} qSq^{T}& =  q_{h}\left( q_{h}(s-1)+1\right) +\left( 1-q_{h}\right) =1-q_{h}^{2}(1-s), \end{aligned}$$

45 $$\begin{aligned} qS\cdot Pq^{T}& =  q_{h}\left( \frac{1}{2}sq_{h}+1-q_{h}\right) +\frac{1}{2} (1-q_{h})^{2}=\frac{1}{2}\left( 1-q_{h}^{2}(1-s)\right) . \end{aligned}$$

Note that $qS\cdot Pq^{T}=qSq^{T}/2$ which simplifies (41) to

46 $$\begin{aligned} \dot{n}=n\left( qSq^{T}\left( \frac{1}{2}W\left( 1-\frac{n}{K}\right) +1\right) -1\right) . \end{aligned}$$

The bracketed terms of (40) are

47 $$\begin{aligned} V_{i}-\sum _{j}q_{j}V_{j}& =  \left( e_{1}S\cdot Pq^{T}-qS\cdot Pq^{T}\right) = \frac{1}{2}\left( 1-q_{h}\right) \left[ q_{h}\left( s-1\right) +1)\right] , \end{aligned}$$

48 $$\begin{aligned} s_{i}-\sum _{j}q_{j}s_{j}& =  (e_{1}Sq^{T}-qSq^{T})=(s-1)q_{h}\left( 1-q_{h}\right) . \end{aligned}$$

After calculations and the substitution $d=1-s$ the following equations for the Hawk proportion $q_{h}$ and for the population size are obtained:

49 $$\begin{aligned} \dot{q}_{h}& =  q_{h}\left( 1-q_{h}\right) \left( \frac{1}{2}W\left( 1-q_{h}d\right) \left( 1-\dfrac{n}{K}\right) -q_{h}d\right) , \end{aligned}$$

50 $$\begin{aligned} \dot{n}& =  n\left( \frac{1}{2}W \left( 1-q_{h}^{2}d\right) \left( 1-\frac{n}{K} \right) -q_{h}^{2}d\right) . \end{aligned}$$

Thus we have embedded the focal game into the replicator dynamics. We should add the background fitness components represented by the background vital rate $\Phi \left( 1-\frac{n}{K}\right) -\Psi$ into the population size equation. Thus we obtain the model derived according to the system (6, 7):

51 $$\begin{aligned} \dot{q}_{h}& =  q_{h}\left( 1-q_{h}\right) \left( \frac{1}{2}W\left( 1-q_{h}d\right) \left( 1-\dfrac{n}{K}\right) -q_{h}d\right) ,\end{aligned}$$

52 $$\begin{aligned} \dot{n}& =  n\left( \left( \Phi +\frac{1}{2}W\left( 1-q_{h}^{2}d\right) \right) \left( 1-\frac{n}{K}\right) -q_{h}^{2}d-\Psi \right) . \end{aligned}$$

The zeros of the right-hand sides of the above equations will give nullclines constituting the equilibria of selection and ecological subsystems. Two rest points of this system are $q_{h}=0$ and 1. A nontrivial rest point, which becomes the frequency nullcline consisting of Nash equilibria, is given by

53 $$\begin{aligned} \tilde{q}_{h}(n)=\dfrac{\frac{1}{2}W\left( 1-\dfrac{n}{K}\right) }{d\left( \frac{1}{2}W\left( 1-\dfrac{n}{K}\right) +1\right) }. \end{aligned}$$

There is a stationary population size at either $\tilde{n}=0$ or at the following positive restpoint which is conditional on the Hawk strategy frequency (describing the attracting density nullcline parametrized by $q_{h}$),

54 $$\begin{aligned} \tilde{n}(q_{h})=K\left( 1-\dfrac{q_{h}^{2}d+\Psi }{\frac{1}{2}W\left( 1-q_{h}^{2}d\right) +\Phi }\right) . \end{aligned}$$

To obtain the model derived according to the payoff-based background fitness we should multiply mortality payoffs by $\left[ W_{b}\left( 1-\frac{n}{K} \right) +s_{b}\right]$ as in (23) and (24). In equations (49) this will be the last term $q_{h}d$ in the long bracket, due to (48), and in Eq. (50) this will be term $qSq^{T}$ in (41) which becomes 1 in the internal bracket in (46). This leads to the model derived according to system (9, 10) :

55 $$\begin{aligned} \dot{q}_{h}& =  q_{h}\left( 1-q_{h}\right) \left( \frac{1}{2}W\left( 1-q_{h}d\right) \left( 1-\dfrac{n}{K}\right) -q_{h}d\left[ W_{b}\left( 1- \frac{n}{K}\right) +s_{b}\right] \right) , \end{aligned}$$

56 $$\begin{aligned} \dot{n}& =  n\left( \left( 1-q_{h}^{2}d\right) \left( \left[ \frac{1}{2}W+W_{b} \right] \left( 1-\frac{n}{K}\right) +s_{b}\right) -1\right) , \end{aligned}$$

leading to the system used in Argasinski and Broom (2012). Then the attracting nullclines representing the equilibria of selection dynamics and ecological dynamics will be

57 $$\begin{aligned} \tilde{q}_{h}(n)& =  \frac{\frac{1}{2}W\left( 1-\dfrac{n}{K}\right) }{d\left( \left( \frac{1}{2}W+W_{b}\right) \left( 1-\frac{n}{K}\right) +s_{b}\right) ,} \end{aligned}$$

58 $$\begin{aligned} \tilde{n}(q_{h})& =  K\left( 1-\frac{1-\left( 1-q_{h}^{2}d\right) s_{b}}{\left( 1-q_{h}^{2}d\right) \left[ \frac{1}{2}W+W_{b}\right] }\right) . \end{aligned}$$

## References

[CR1] Antal T, Traulsen A, Ohtsuki H, Tarnita CE, Nowak MA (2009). Mutation-selection equilibrium in games with multiple strategies. J Theor Biol.

[CR2] Argasinski K (2006). Dynamic multipopulation and density dependent evolutionary games related to replicator dynamics. A metasimplex concept. Math Biosci.

[CR3] Argasinski K (2012). The dynamics of sex ratio evolution dynamics of global population parameters. J Theor Biol.

[CR4] Argasinski K (2013). The dynamics of sex ratio evolution: from the gene perspective to multilevel selection. PloS One.

[CR001] Argasinski K (2017) The dynamics of sex ratio evolution: the impact of males as passive gene carriers on multilevel selection. Dyn Games Appl **(to appear)**

[CR5] Argasinski K, Broom M (2012). Ecological theatre and the evolutionary game: how environmental and demographic factors determine payoffs in evolutionary games. J Math Biol.

[CR6] Argasinski K, Broom M (2013). The nest site lottery: how selectively neutral density dependent growth suppression induces frequency dependent selection. Theor Popul Biol.

[CR002] Argasinski K, Broom M (2013). Evolutionary stability under limited population growth: eco-evolutionary feedbacks and replicator dynamics. Ecol Complex.

[CR7] Argasinski K, Kozłowski J (2008). How can we model selectively neutral density dependence in evolutionary games. Theor Pop Biol.

[CR003] Birch LC (1960). The genetic factor in population ecology. Am Nat.

[CR8] Bowers RG, White A, Boots M, Geritz SA, Kisdi E (2003). Evolutionary branching/speciation: contrasting results from systems with explicit or emergent carrying capacities. Evol Ecol Res.

[CR9] Brandon RN, Ramsey D, Hull D, Ruse M (2007). What’s wrong with the emergentist statistical interpretation of natural selection and random drift?. Cambridge companion to the philosophy of biology.

[CR10] Broom M (2002). A unified model of dominance hierarchy formation and maintenance. J Theor Biol.

[CR11] Broom M, Cannings C (2002). Modelling dominance hierarchy formation as a multi-player game. J Theor Biol.

[CR12] Broom M, Ruxton GD (1998). Evolutionarily stable stealing: game theory applied to kleptoparasitism. Ann Hum Genet.

[CR13] Broom M, Rychtar J (2013). Game-theoretical models in biology.

[CR14] Bürger R (2000). The mathematical theory of selection, recombination, and mutation.

[CR15] Caswell H (2001). Matrix population models.

[CR16] Cannings C, Lessard S (2012). Topics in the theory of ESS’s. Mathematical and statistical developments of evolutionary theory. Lecture Notes in mathematics.

[CR17] Chakra MA, Hilbe C, Traulsen A (2014). Plastic behaviors in hosts promote the emergence of retaliatory parasites. Sci Rep.

[CR18] Claussen JC, Traulsen A (2005). Non-Gaussian fluctuations arising from finite populations: exact results for the evolutionary Moran process. Phys Rev E.

[CR19] Cressman R, Dash AT, Akin E (1986). Evolutionary games and two species population dynamics. J Math Biol.

[CR20] Cressman R (1992). The stability concept of evolutionary game theory.

[CR21] Cressman R, Garay J (2003). Evolutionary stability in Lotka–Volterra systems. J Theor Biol.

[CR22] Cressman R, Garay J (2003). Stability in N-species coevolutionary systems. Theor Popul Biol.

[CR23] Cressman R, Garay J, Hofbauer J (2001). Evolutionary stability concepts for N-species frequency-dependent interactions. J Theor Biol.

[CR24] Cressman R, Křivan V (2010). The ideal free distribution as an evolutionarily stable state in density-dependent population games. Oikos.

[CR49] Crow, JF, Kimura M (1970) An introduction to population genetics theory. An introduction to population genetics theory, Harper and Row

[CR25] Dawkins R (1989). The selfish gene.

[CR26] Dall SR, Houston AI, McNamara JM (2004). The behavioural ecology of personality: consistent individual differences from an adaptive perspective. Ecol Lett.

[CR27] Dercole F, Rinaldi S (2008). Analysis of evolutionary processes: the adaptive dynamics approach and its applications: the adaptive dynamics approach and its applications.

[CR28] Dieckmann U, Law R (1996). The dynamical theory of coevolution: a derivation from stochastic ecological processes. J Math Biol.

[CR29] Doebeli M, Hauert C, Killingback T (2004). The evolutionary origin of cooperators and defectors. Science.

[CR30] Fu F, Wang L, Hauert C, Nowak MA (2009). Evolutionary dynamics on graphs: efficient method for weak selection. Phys Rev E.

[CR31] Geritz SA, Kisdi É, Meszéna G, Metz JAJ (1998). Evolutionarily singular strategies and the adaptive growth and branching of the evolutionary tree. Evol Ecol.

[CR32] Geritz SA, Kisdi E (2012). Mathematical ecology: why mechanistic models?. J Math Biol.

[CR33] Gerlee P, Lundh T (2010). Rock-scissor-paper dynamics in a digital ecology. In: Proceedings of the twelfth international conference on the synthesis and simulation of living systems, pp 285–295

[CR004] Ginzburg LR (1983). Theory of natural selection and population growth.

[CR34] Gokhale C, Hauert C (2016). Eco-evolutionary dynamics of social dilemmas. Theor Popul Biol.

[CR35] Gokhale C, Traulsen A (2010). Evolutionary games in the multiverse. PNAS.

[CR36] Grimm V, Railsback SF (2005). Individual-based modeling and ecology.

[CR37] Haigh J (2002). Probability models.

[CR38] Hartl DL, Clark AG (1997). Principles of population genetics.

[CR39] Hauert C, Holmes M, Doebeli M (2006). Evolutionary games and population dynamics: maintenance of cooperation in public goods games. Proc R Soc B Biol Sci.

[CR40] Hauert C, Wakano JY, Doebeli M (2008). Ecological public goods games: cooperation and bifurcation. Theor Popul Biol.

[CR41] Hauser OP, Traulsen A, Nowak MA (2014). Heterogeneity in background fitness acts as a suppressor of selection. J Theor Biol.

[CR42] Hofbauer J, Sigmund K (1988). The theory of evolution and dynamical systems.

[CR43] Hofbauer J, Sigmund K (1998). Evolutionary games and population dynamics.

[CR44] Horan BL (1994). The statistical character of evolutionary theory. Philos Sci.

[CR45] Houston AI, McNamara JM (1991). Evolutionarily stable strategies in the repeated hawk–dove game. Behav Ecol.

[CR46] Houston AI, McNamara JM (2005). John Maynard Smith and the importance of consistency in evolutionary game theory. Biol Phil.

[CR47] Huang W, Hauert C, Traulsen A (2015). Stochastic game dynamics under demographic fluctuations. PNAS.

[CR48] Hui C (2006). Carrying capacity, population equilibrium, and environment’s maximal load. Ecol Model.

[CR005] Hutchinson GE (1965) The ecological theater and the evolutionary play. Yale University Press

[CR50] Kimura M (1968). Evolutionary rate at the molecular level. Nature.

[CR51] Křivan V (1998). Effects of optimal antipredator behavior of prey on predator-prey dynamics: the role of refuges. Theor Popul Biol.

[CR52] Křivan V (2003). Competitive co-existence caused by adaptive predators. Evol Ecol Res.

[CR53] Křivan V (2013). The Allee-type ideal free distribution. J Math Biol.

[CR54] Łomnicki A (1988). Population ecology of individuals.

[CR60] Lunel, SM (Eds.) Stochastic and spatial structures of dynamical systems. North-Holland, pp 183–231

[CR55] Matthen M, Ariew A (2002). Two ways of thinking about fitness and natural selection. J Philos.

[CR56] Matthen M, Ariew A (2009). Selection and causation. Philos Sci.

[CR57] Maynard Smith J (1982). Evolution and the theory of games.

[CR58] McNamara JM (2013). Towards a richer evolutionary game theory. J R Soc Interface.

[CR59] Metz JAJ, Geritz SA, Meszéna G, Jacobs FJA, van Heerwaarden JS (1996) Adaptive dynamics, a geometrical study of the consequences of nearly faithful reproduction. In: van Strien SJ, Verduyn Lunel SM (eds) Stochastic and spatial structures of dynamical systems. North-Holland, Amsterdam, pp 193–231

[CR61] Metz JAJ, Jørgensen SE, Fath BD (2008). Fitness. Evolutionary ecology. Encyclopedia of ecology.

[CR62] Mills SK, Beatty JH (1979). The propensity interpretation of fitness. Philos Sci.

[CR63] Morris DW (2011). Adaptation and habitat selection in the eco-evolutionary process. Proc R Soc B.

[CR006] Mylius SD (1999). What pair formation can do to the battle of the sexes: towards more realistic game dynamics. J Theor Biol.

[CR64] Novak S, Chatterjee K, Nowak MA (2013). Density games. J Theor Biol.

[CR65] Nowak MA, Roch S (2007). Upstream reciprocity and the evolution of gratitude. Proc R Soc Biol Sci.

[CR66] Nowak MA, Sasaki A, Taylor C, Fudenberg D (2004). Emergence of cooperation and evolutionary stability in finite populations. Nature.

[CR67] Ohta T (2002). Near-neutrality in evolution of genes and gene regulation. Proc Natl Acad Sci.

[CR68] Ohtsuki H, Hauert C, Lieberman E, Nowak MA (2006). A simple rule for the evolution of cooperation on graphs and social networks. Nature.

[CR69] Orr HA (2009). Fitness and its role in evolutionary genetics. Nat Rev Genet.

[CR70] Pelletier F, Garant D, Hendry AP (2009). Eco-evolutionary dynamics. Phil Trans R Soc B.

[CR71] Pena J, Pestelacci E, Berchtold A, Tomassini M (2011). Participation costs can suppress the evolution of upstream reciprocity. J Theor Biol.

[CR72] Post DM, Palkovacs EP (2009). Eco-evolutionary feedbacks in community and ecosystem ecology: interactions between the ecological theatre and the evolutionary play. Phil Trans R Soc B.

[CR73] Ramsey G (2013). Can fitness differences be a cause of evolution?. Philos Theor Biol.

[CR74] Roff DA (2008). Defining fitness in evolutionary models. J Genet.

[CR75] Rosenberg A, Williams M (1986). Fitness as primitive and propensity. Philos Sci.

[CR76] Schoener TW (2011). The newest synthesis: understanding the interplay of evolutionary and ecological dynamics. Science.

[CR77] Sieber M, Malchow H, Hilker FM (2014). Disease-induced modification of prey competition in eco-epidemiological models. Ecol Complex.

[CR78] Tarnita CE, Antal Nowak MA (2009). Mutation-selection equilibrium in games with mixed strategies. J Theor Biol.

[CR79] Taylor PD, Day T, Wild G (2007). Evolution of cooperation in a finite homogeneous graph. Nature.

[CR80] Taylor C, Fudenberg D, Sasaki A, Nowak MA (2004). Evolutionary game dynamics in finite populations. B Math Biol.

[CR81] Taylor C, Nowak MA (2006). Evolutionary game dynamics with non-uniform interaction rates. Theor Pop Biol.

[CR82] Traulsen A, Hauert C (2009). Stochastic evolutionary game dynamics. Rev Nonlinear Dyn Complex.

[CR83] Uchmański J, Grimm V (1996). Individual-based modelling in ecology: what makes the difference?. Trends Ecol Evol.

[CR84] Upadhyay SK (2006). Chemical kinetics and reaction dynamics.

[CR85] Voelkl B (2010). The ‘Hawk–Dove’ game and the speed of the evolutionary process in small heterogeneous populations. Games.

[CR86] Walsh DM (2010). Not a sure thing: fitness, probability, and causation. Philos Sci.

[CR87] Wild G, Traulsen A (2007). The different limits of weak selection and the evolutionary dynamics of finite populations. J Theor Biol.

[CR88] Williams GC (1996). Plan and purpose in nature.

[CR89] Wolf M, McNamara JM (2012). On the evolution of personalities via frequency-dependent selection. Am Nat.

[CR90] Wolf M, Van Doorn GS, Leimar O, Weissing FJ (2007). Life-history trade-offs favour the evolution of animal personalities. Nature.

[CR91] Wolf M, Weissing FJ (2010). An explanatory framework for adaptive personality differences. Phil Trans Proc R Soc B.

[CR92] Wolf M, Weissing FJ (2012). Animal personalities: consequences for ecology and evolution. Trends Ecol Evol.

[CR93] Wu B, Altrock PM, Wang L, Traulsen A (2010). Universality of weak selection. Phys Rev E.

[CR94] Zhang F, Hui C (2011). Eco-evolutionary feedback and the invasion of cooperation in prisoner’s dilemma games. PLoS One.

